# Supplementary material for: Time‑resolved multi-omic analysis of paclitaxel exposure in human iPSC‑derived sensory neurons unveils mechanisms of chemotherapy‑induced peripheral neuropathy
Source: Cell Death Dis. 2026 Feb 10;17(1):211. doi: 10.1038/s41419-026-08445-2 (PMC12921266; doi:10.1038/s41419-026-08445-2)
Supplement: Supplementary file 15 — Dataset 1. [file 41419_2026_8445_MOESM15_ESM.zip › CDD_Open_Data/04_RNA_Seq/Timeline_panel_plots_etc_2023-01-23_1609.zip!/Panel_Plots_etc/Evidence_Plots/6h/evidence_plot_standard contrasts_msigdb_go_bp_6h_PTX_vs_DMSO_ID2_M22379_GOBP_MITOCHONDRIAL_ELECTRON_TRANSPORT_NADH_TO_UBIQUINON.pdf]

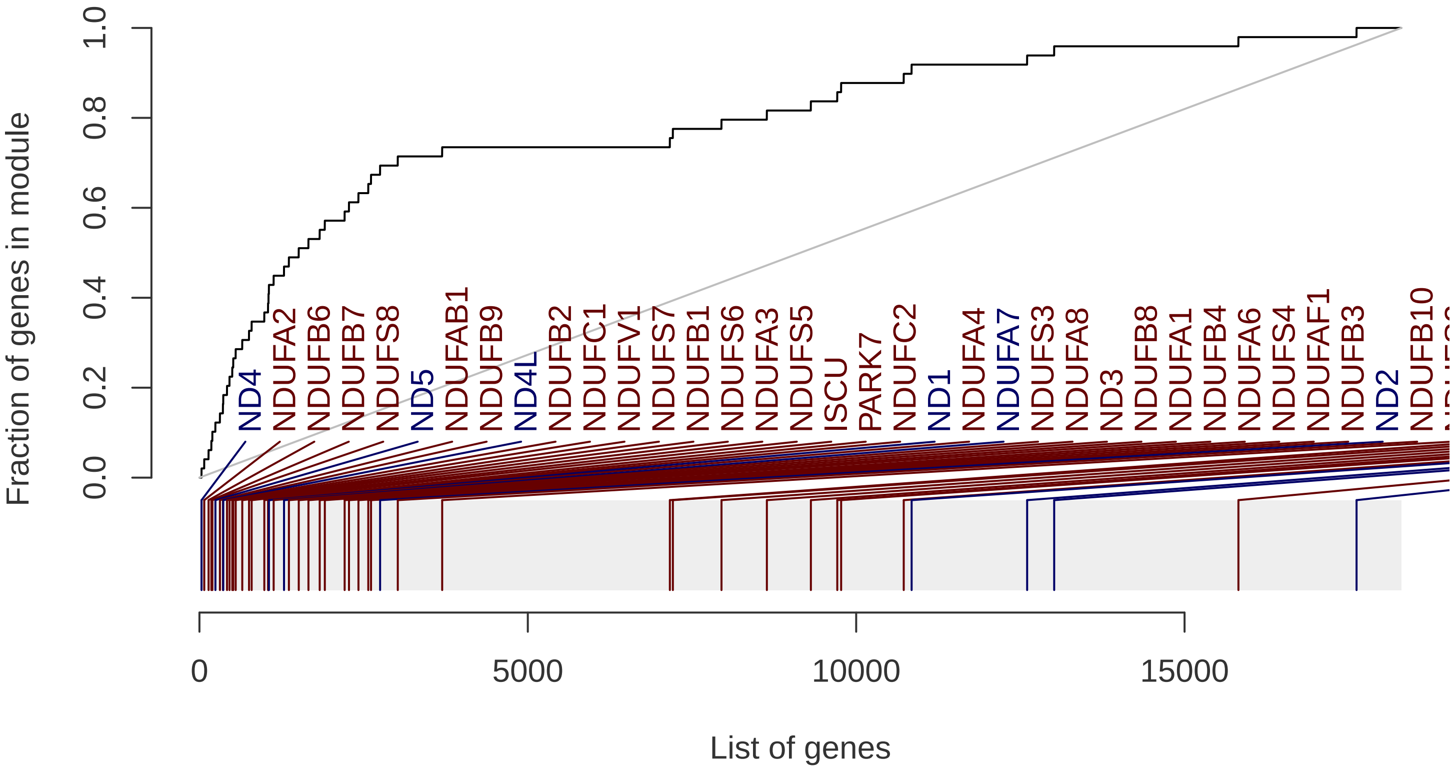

Fraction of genes in module
0.0
0.2
0.4
0.6
0.8
1.0
ND4
NDUFA2
NDUFB6
NDUFB7
NDUFS8
ND5
NDUFAB1
NDUFB9
ND4L
NDUFB2
NDUFC1
NDUFV1
NDUFS7
NDUFB1
NDUFS6
NDUFA3
NDUFS5
ISCU
PARK7
NDUFC2
ND1
NDUFA4
NDUFA7
NDUFS3
NDUFA8
ND3
NDUFB8
NDUFA1
NDUFB4
NDUFA6
NDUFS4
NDUFAF1
NDUFB3
ND2
NDUFB10
0
5000
10000
15000
List of genes
